# A Novel Preprocessing Method Using Hilbert Huang Transform for MALDI-TOF and SELDI-TOF Mass Spectrometry Data

**DOI:** 10.1371/journal.pone.0012493

**Published:** 2010-08-31

**Authors:** Li-Ching Wu, Hsin-Hao Chen, Jorng-Tzong Horng, Chen Lin, Norden E. Huang, Yu-Che Cheng, Kuang-Fu Cheng

**Affiliations:** 1 Graduate Institute of System Biology and Bioinformatics, National Central University, Jhongli, Taiwan; 2 Research Center for Biotechnology and Biomedical Engineering, National Central University, Jhongli, Taiwan; 3 Department of Computer Science and Information Engineering, National Central University, Jhongli, Taiwan; 4 Department of Bioinformatics, Asia University, Wu-feng, Taiwan; 5 Research Center for Adaptive Data Analysis, National Central University, Jhongli, Taiwan; 6 Proteomics Laboratory, Cathay Medical Research Institute, Cathay General Hospital, Xizhi, Taiwan; 7 Graduate Institute of Statistics, National Central University, Jhongli, Taiwan; 8 Graduate Institute of Statistics, China Medical University, Taichung, Taiwan; Queen Elizabeth Hospital, Hong Kong

## Abstract

**Motivation:**

Mass spectrometry is a high throughput, fast, and accurate method of protein analysis. Using the peaks detected in spectra, we can compare a normal group with a disease group. However, the spectrum is complicated by scale shifting and is also full of noise. Such shifting makes the spectra non-stationary and need to align before comparison. Consequently, the preprocessing of the mass data plays an important role during the analysis process. Noises in mass spectrometry data come in lots of different aspects and frequencies. A powerful data preprocessing method is needed for removing large amount of noises in mass spectrometry data.

**Results:**

Hilbert-Huang Transformation is a non-stationary transformation used in signal processing. We provide a novel algorithm for preprocessing that can deal with MALDI and SELDI spectra. We use the Hilbert-Huang Transformation to decompose the spectrum and filter-out the very high frequencies and very low frequencies signal. We think the noise in mass spectrometry comes from many sources and some of the noises can be removed by analysis of signal frequence domain. Since the protein in the spectrum is expected to be a unique peak, its frequence domain should be in the middle part of frequence domain and will not be removed. The results show that HHT, when used for preprocessing, is generally better than other preprocessing methods. The approach not only is able to detect peaks successfully, but HHT has the advantage of denoising spectra efficiently, especially when the data is complex. The drawback of HHT is that this approach takes much longer for the processing than the wavlet and traditional methods. However, the processing time is still manageable and is worth the wait to obtain high quality data.

## Introduction

Mass spectrometry is currently used to explore protein profiles expressed under different physiological and pathophysiological conditions [1]. Moreover, recent progress has opened up new avenues for tumor-associated biomarker discovery [2]. A mass spectrum of a sample is a profile representing the distribution of components by mass-to-charge ratio. Spectra of tissues or fluids, like serum, are studied for possible profile changes that further disease diagnosis. Matrix assisted laser desorption ionization (MALDI) and surface-enhanced laser desorption ionization (SELDI) time of flight (TOF) are the two commonly techniques used to generate profiles from experimental samples. The chief feature of mass spectra is the peaks detected in terms of their intensity values and time of flight values. Further peak identification can be done if tandem mass spectrometry is available [3]. Since each spectrum contains ten thousands of time of flight points with various intensities, noise in the spectra is unavoidable. Therefore, it is important to develop a suitable algorithm for data preprocessing that improves performance when analyzing spectra.

Recently, various data preprocessing methods have been used and these usually comprise several steps. First, baseline subtraction is often used to rescale the plots with the aims of removing systematic artifacts produced by small clusters of matrix material [4]. Next, denoising attempts to remove noise signals that are added to the true spectra from the matrix material and by sample contaminants (chemical noise) together with noise caused by the physical characteristics of the machine (electrical noise) [5], [6]. Furthermore, alignment is a required for combining unusual groups of data together. The same peak may be present, but with small gaps between the different biological samples due to unavoidable inaccuracy in the spectrum. Peak detection is still necessary across every method and is a key feature of preprocessing the data. It is necessary to detect each peak by relying on their peak intensity and time of flight. Finally, normalization helps us to have a uniform format for the analysis of the data and this corrects any systematic variation between the different spectra [7].

There are many studies that have described preprocessing of mass spectrum data [6], [8], [9], [10], [11], [12], [13] and have explored their approach's influence on the raw data. In the study of Meuleman, Engwegen et al. [14], they compared various different algorithms that can be used for normalization. In another, Beyer, Walter et al. [15] compared the performance of the package “Ciphergen Express Software 3.0”, which is produced by Ciphergen against the “R package PROcess”. Recently, Cruz-Marcelo, Guerra et al. [7] compared a number of widely used algorithms, namely “ProteinChip© Soft-ware 3.1” (Ciphergen Biosystems), “Biomarker” Wizard (Ciphergen Biosystems), “PROcess”, which was written by Xiaochun Li as the “BioConductor” package, “Cromwell” written using Matlab scripts, “SpecAlign” developed by Wong, Cagney et al. [11], and “MassSpecWavelet” developed by Du, Kibbe et al. [12] as a “BioConductor” package. Nevertheless, although many preprocessing methods have been put forward, the preprocessing algorithm can still be improved.

In the past, the scientists have tried to compute a formula for noise that consists of chemical and machine noise using a statistical method and then constructing a model based on this. However, the chemical noise is generally due to true peaks, namely organic acids, which are part of the matrix used in mass spectrometry. The matrix has two purposes: ionization and protection. It provides hydrogen ions to the peptides or proteins, which are then allowed to undergo ionization and flight in the machine. In addition, the matrix protects the peptide or protein during the laser flash. Matrix noise usually appears in the low mass-to-charge ratio regions (<1000DA). Nonetheless, we need to understand that peaks in the low mass-to-charge ratio region are not only due to chemical noise but also contain true signal peaks. If we mixed the chemical noise with the machine noise as part of preprocessing, we might conclude that the noise strongly affecting the low mass-to-charge ratio region is due to the abundance of organic acids in this region. However, if we take into account the difference between chemical and machine noise when we analyze the spectra, we ought to be able to separate chemical noise from machine noise; this is because the peaks in the low mass-to-charge ratio regions are due to the organic acids and thus distinct from machine noise. The machine noise may come from variety of different sources includes air dust, electric detection limitation, electric white noise, and even earth magnetic field. These noises may not have fixed frequences since the m/z value have measurement shifting problem.

In the present study we present a novel preprocessing method using Hilbert Huang transformation (HHT) that is used to decompose a non-linear and non-stationary model. By using HHT, the data can be decomposed into different trends which separate some noises from signals. The main advantage of HHT is non-stationary. It does not make strong assumption that the signal respond to the axis to be stationary distributed. Since the m/z axis exist shifting problem, the HHT can eliminate more non-stationary noises than stationary method such as wavelet. The disadvantage is the calculation time will much longer then stationary method. We then compare our algorithm with three familiar preprocessing methods and with another algorithm that has been suggested by Cruz-Marcelo, Guerra et al. [7].

## Materials and Methods

### Hilbert Huang transformation

HHT [16] is an adaptive data analysis method for non-linear and non-stationary processes. We use HHT to define the trends in a spectrum. In the past, we have defined the trend that represents the baseline and the noise as a straight line, which is then fitted to the spectrum; then we removed the straight line to yield a zero-mean residue. However, such trends are not suitable for non-linear data and the real-world. Noise exists non-linearly and is non-stationary. In reality, the line is non-linear and non-stationary when we try to rescale spectra.

The main feature of the HHT is the empirical mode decomposition (EMD) method with which any complicated data can be decomposed into a finite and often a small number of components called intrinsic mode functions (IMF). We define the IMF if the intrinsic mode of oscillation satisfies two conditions: firstly that the number of the extrema and the number of the zero-crossings must either equal or differ at most by one in the whole dataset and, secondly, the mean value of the envelope defined by the local minima is zero at any point. The IMFs by the EMD method are chiefly obtained by an approach called the sifting process. Actually, the number of IMFs is closed to log2N where N denotes the total number of data points. The sum of all IMFs is equal to the original data.

**Figure 1:**
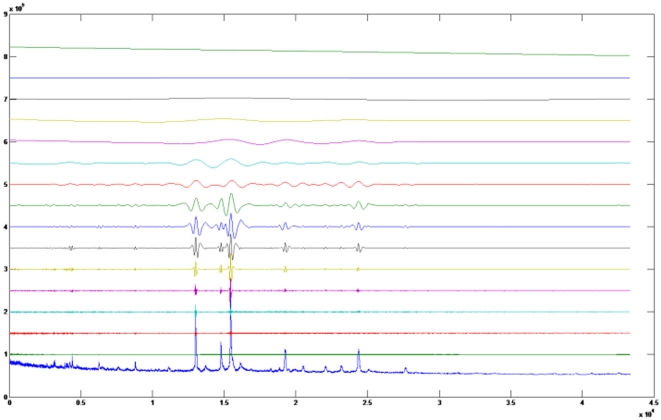
Components decomposed using HHT. HHT decomposes the spectrum into sixteen components. From bottom to top, we call them as C1, C2, and so on. Summation of all components can is the original spectrum.

We chose one of the ovarian cancer datasets from the National Cancer Institute published by Kwon, Vannucci et al. [6] to undergo the HHT process. Sixteen IMF components were identified while applying sifting process to our data. As is shown in the Figure 1, the later components, namely the ones from the fourteenth IMF to the sixteenth IMF, can be removed for the purpose of rescaling; we also removed the components from first IMF to sixth IMF for the purpose of denoising. Thus a significant part of the chemical noise can be separated from the main spectrum by removing the first components.

### Subsequent Modifications

**Figure 2:**
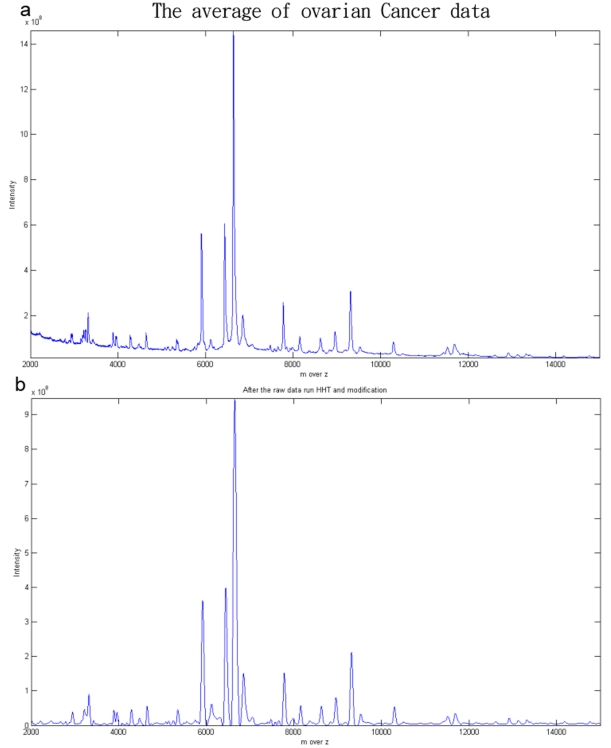
Before and after HHT preprocessing. (a) The average of fifty ovarian cancer datasets. There are greater amounts of noise in the low region than in the high region. The scale is approximately ten to the ninth power. (b) The same figure after using the Hilbert Huang transformation formula and the various modifications carried out after preprocessing. When comparing with (a), it can be seen that the chief peaks and the profile are maintained.

In addition to using the HHT for de-noising, the baseline needs to be adjusted. Here we apply SpecAlign software for baseline estimation, which is available at PHYSCHEM.OX.AC.UK/∼JWONG/SPECALIGN [11]. For removing the baseline, the software has two user-defined options: window size of the baseline and subtraction of the baseline. We set the window size as 20, and then we remove the baseline. After baseline subtraction, we rescaled the spectrum to positive. We moved the whole spectrum to be positive by changing the intensity values. However, we did not change other parameters. Our method, which we have called HHTMass, consists of using the Hilbert Huang transformation for denoising followed by modification of the spectrum by baseline subtraction and rescaling. The spectrum before and after the HHT preprocessing are shown in Figure 2(a) and 2(b) (spectrum source shown in data source section).

### Peak detection

We apply three methods, namely MassSpecWavelet [12], SpecAlign [11], and PROcess [9] for peak detection. The major feature of MassSpecWavelet is that the package does not contain any preprocessing method. According to Cruz-Marcelo, Guerra et al. [7], MassSpecWavelet has the best performance in terms of peak detection. PROcess is a BioConductor package by Li [9], which has high quality of peak quantification. SpecAlign, written by Wong [11], is a well known spectrum analysis software package; it has the useful property of containing many user defined options that increase choice. The preprocessing methodology linked to MassSpec-Wavelet was designated as HHTMass1, that linked to SpecAlign as HHTMass2, and that linked to PROcess as HHTMass3.

### Data source

Two different samples are used in this study. Ovarian cancer data is acquired from the authors of [6] in National Cancer Institute. Serum samples from women diagnosed with ovarian cancer and women hospitalized for other conditions were collected at the Mayo Clinic from 1980 to 1989. The dataset was analyzed by SELDI-TOF MS using the CM10 chip type [17]. The ProteinChip Biomarker System was used for protein expression profiling.

The spectrum has two properties, m over z and intensity. The dataset consists of fifty samples after 1986. The m over z ranges are between 58Da and 101453Da. The intensity ranges are between −3.27E7 and 2.14E9. Based on the results of Cruz-Marcelo, Guerra et al. et al. [7] and Kwon, Vannucci et al. [6], in this study we examined the m over z range from 2000Da to 15000Da. Each sample has 21552 points.

**Figure 3:**
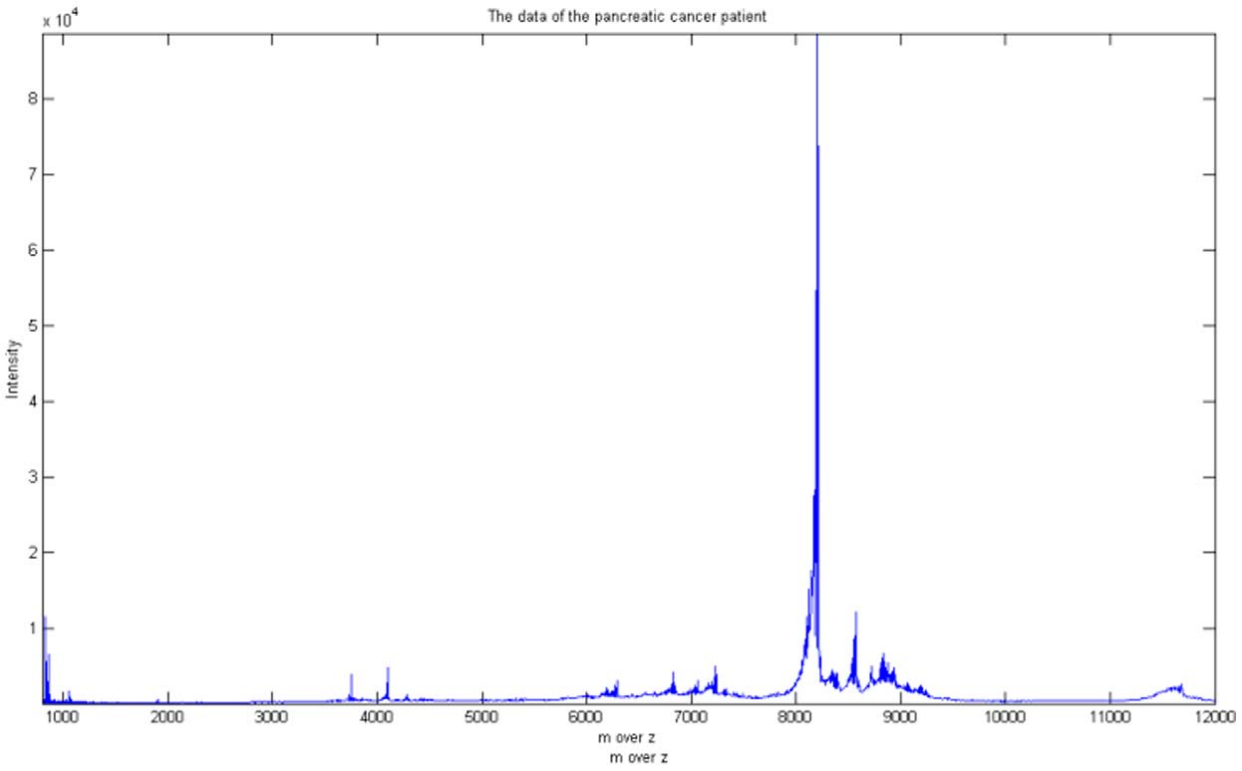
Sample pancreatic cancer data. Original data of pancreatic cancer provided by Ge and Wong (2008). In this dataset, there is more noise where the peaks exist.

As we see the **Figure 2(a)**, the spectrum is full of noise, especially in low m over z value region. Several different denoising methods have been developed to handle this type of data [6], [13], [18], [19]. In general, these approaches use a simulated model for comparing the performance of preprocessing methods. However, the above preprocessing methods seem to be pre-justified in their model. The real distribution of noise is more irregular in real experiments because we cannot understand completely how electrical and chemical noise is generated in a spectrum. Therefore, in this study we compared our approach with the other methods using real data rather than simulated data. It is well-known that Morris, et al. [20] proposed a model using Gaussian white noise and that Cruz-Marcelo, Guerra et al. [7] proposed the ARMA model. However, there is no evidence to show which model fits real data. The preprocessing methods proposed up to now fit their data and their model. For example, we requested a pancreatic cancer dataset from [21] and compared it to the ovarian cancer data. In the **Figure 3**, the pancreatic cancer spectrum has high amounts of noise in the regions where peaks exist, which is different from [6], where the noise is greater in the low region. Therefore, approaches to constructing the model cannot be uniform. In this context then it is clear that real data is the best target when carrying out comparisons.

In fact, while processing the mass spectrum data, we substituted the scale of time of flight for the scale of m/z values. The transform was carried out according to the formula (1).
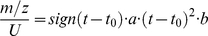
(1)


Here t denotes the time of flight, U = 25000, a = 3.36E8, b = 0.00235, and t0 = 3.7071E-7. A single spectrum has 21 551data points in our experiments [6]. After the above preprocessing step, we should be able to compare the spectra and distinguish biomarkers that identify the differences between healthy and diseased individuals.

### Methods of comparison

**Figure 4:**
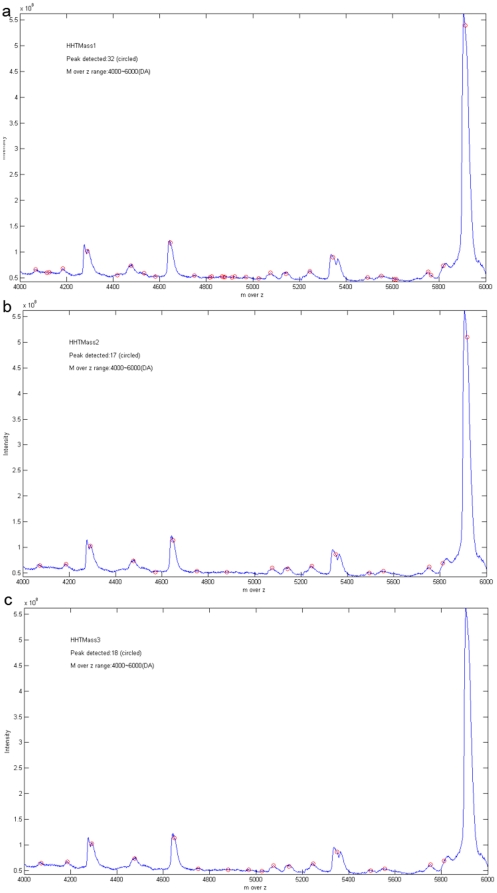
Different peaks detected by different peak selection method. (a) The ovarian cancer dataset preprocessed by HHTMass1. The chosen area is from 4000DA to 6000DA. The figure is the original spectrum and the circled points are the peaks detected by HHTMass1. HHTMass1 detected 32 peaks in this region. (b) The ovarian cancer dataset preprocessed by HHTMass2. The chosen area is from 4000DA to 6000DA. The figure is the original spectrum and the circled points are the peaks detected by HHTMass2. HHTMass1 detected 17 peaks in this region. (c) The ovarian cancer dataset preprocessed by HHTMass3. The chosen area is from 4000DA to 6000DA. The figure is the original spectrum and the circled points are the peaks detected by HHTMass3. The largest peak in the region between 5000DA and 6000DA was not detected by HHTMass3. HHTMass3 detected 18 peaks in this region.

Previously, scientists have compared performance mainly in terms of peak detection and peak quantification. Peak detection means that we compare the number of detected prevalent peaks between the different preprocessing methods. Peak quantification means the differences in m over z values and the differences in intensity between the detected prevalent peaks and original spectrum are compared. Using the three methods in the present study, the number of detected peaks is quite different. As can be seen in **Figure 4(a)** and **Figure 4(b)**, HHTMass1 detects the most peaks and HHTMass2 detects the least peaks; furthermore, **Figure 4(c)** shows that HHTMass3 failed to detect the biggest peak in the B (4000DA∼6000DA) region. Therefore, it is clear that the number of the peaks detected is not the only reference material that we need to consider when assessing the performance of preprocessing methods and these are explored below.

In this context, we would expect that the profile of the preprocessed spectrum should be similar to that of the original, especially the intensity of the obvious peaks. However, part of preprocessing aims at decreasing the large scale of the spectrum. The scale of the ovarian dataset is very large and is full of complex signals. With such a large scale, noise exists to a very significant extent across the spectrum. The best approach to this problem is to maintain the exterior of the profile; therefore we decreased the scale by about 50% in proportion to the original. **Figure 2(a)** represents the original data and **Figure 2(b)** represents the data after using Hilbert Huang transformation formula and other modifications during preprocessing. When the two figures are compared, it can be seen that the chief peaks and the exterior profile of the spectrum are maintained. Nevertheless, the approach has attempted to remove the signaling errors due to machine and chemical noise. Although we have tried to maintain the exterior profile of the spectrum, we still need to determine whether the peaks present in the preprocessed spectrum are noise or true signals. Finally, when correcting spectra and for visual calibrating purposes, it is better if the baseline of the spectrum is moved to the origin point of the coordinates. Therefore, the final goals of a preprocessing method include retaining the spectrum profile, removing noise, and adjusting the baseline.

Using a comparison of peak numbers is only one way of assessing the performance of a preprocessing method. In this study we not only calculated the number of detected peaks but also assessed the real location of the peaks in the spectrum. When the dataset has a large scale, which is the case with the present dataset, peak quantification as an assessment method needs to be replaced because once we have rescaled the spectrum but maintained the spectrum profile then the relative intensity of the peaks becomes meaningless. Therefore, in this analysis, we used visual comparison as the means of assessing performance rather than peak quantification. In this context, the visual comparison involves comparing the distance between the peaks and appearance of the peaks as detected before and after preprocessing.

Cruz-Marcelo, Guerra et al. [7] suggested several methods of dealing with a spectrum and these included MassSpecWavelet [12] for peak detection and PROcess [9] for peak quantification. Cruz-Marcelo, Guerra et al. [7] suggested that a combined method involving both MassSpecWavelet and PROcess could be used. In addition to the above, we also used the commonly available tool SpecAlign. [11]. Thus, in this analysis, we compare our preprocessing method with those mentioned above and these are abbreviated to SpecA-lign SA, MassSpecWavelet MSW and PROcess PRO as shown in the Result sections.

## Results

We computed the average of fifty original ovarian cancer spectrums and then identified with symbols where the various methods detected peaks using the average raw data. This allows performance to be more obviously compared. Our preprocessing method was then utilized and the best peak detection system identified; then we compare the results of five well known preprocessing methods with our preprocessing approach.

### Results after Hilbert Huang transformation and modification

**Figure 5:**
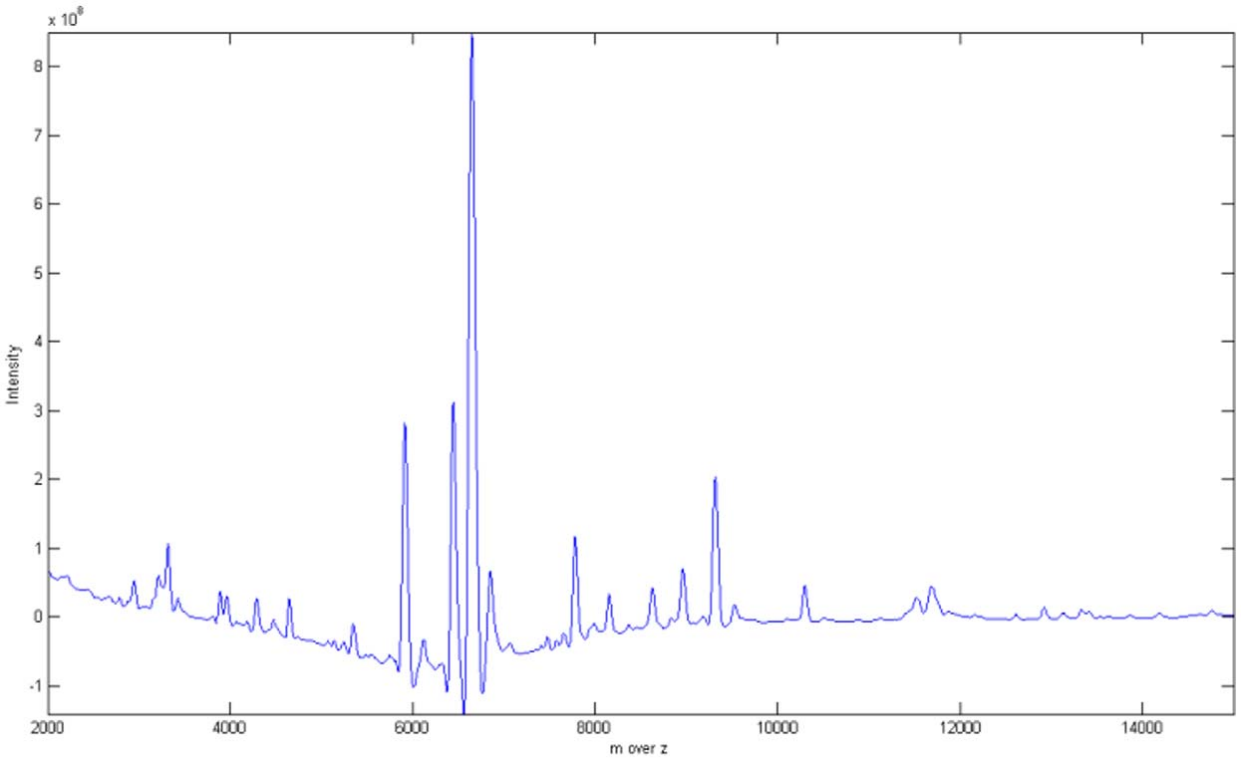
Spectrum data after using HHT preprocessing. The data after using Hilbert Huang transformation without modification. The high frequency noises are removed.

We used HHT to preprocess the ovarian cancer data. The raw data is obviously full of noise, especially in the low region. HHT decomposed each spectrum into sixteen components. We called these components C*i* for *i* [1,2,…,16] (**Figure 1**). Figure 1 indicates that the components from C1 to C7 contain mostly noise, while components from C14 to C16 are associated with the trend of the spectrum. When these components are removed (**Figure 5**) then the wave pattern become much smoother. After HHT, the spectrum is made up of both positive and negative parts. Based on the results of the modification shown in **Figure 2(b)**, we subtract the baseline from spectra and rescale the spectrum to be positive.

### Results of a comparison between our methods

**Figure 6:**
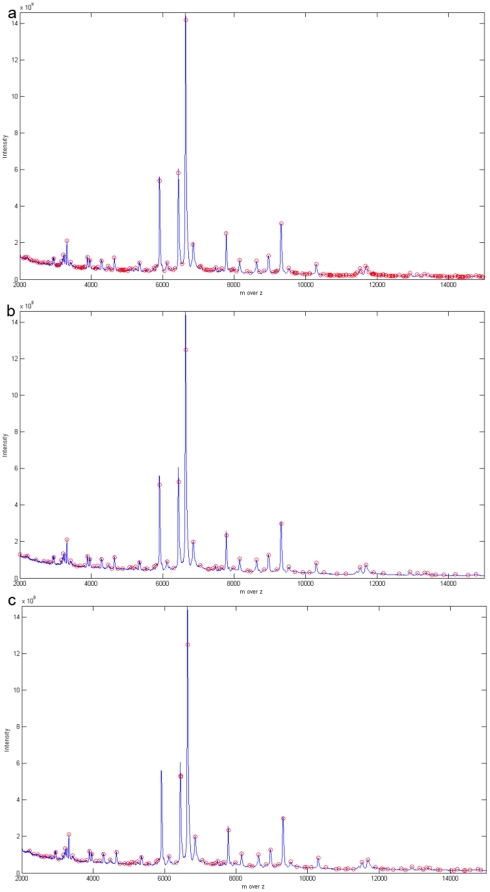
Different peaks detected by different peak selection method on full spectrum us HHTMass. (a) The ovarian cancer dataset preprocessed by HHTMass1. The detected peaks cover most raised peaks. HHTMass1 detected 218 peaks in the whole region. (b) The ovarian cancer dataset preprocessed by HHTMass2. HHTMass2 tends to detect the more obvious peaks. This algorithm detected 80 peaks across the whole region. (c) The ovarian cancer dataset preprocessed by HHTMass3. HHTMass3 misses the third largest peak close to 6000DA. HHTMass3 detected 108 peaks in the whole region.

After HHT and the follow up modifications, we used MassSpecWavelet, SpecAlign, and PROcess for peak detection. It should be note that these algorithms were not used for preprocessing, which was only carried out by HHTMass. We marked an area on the original spectrum and separate this into seven regions for convenience. As shown in **Table 1**, HHTMass1 detected the most number of peaks, namely 218. In contrast, HHTMass2 detected the least number of peaks. Nevertheless, although HHTMass2 (**Figure 6(b)**) detected the least number of peaks, those detected covered all of the obvious peaks in the original spectrum. This differed from HHTMass3, which missed several significant peaks (**Figure 6(c)**). Based on the above results, we chose HHTMass1 and HHTMass2 as our approaches to spectrum analysis and rejected HHTMass3.

**Table 1:** The amount of the peaks detected in our three algorithms.

Algorithm	Total region	Region A	Region B	Region C	Region D	Region E	Region F	Region G
HHTMass1	218	35	32	27	21	44	38	21
HHTMass2	80	18	17	6	11	8	9	3
HHTMass3	108	21	18	19	13	14	13	10

The table shows the results of different preprocessing methods. Region A represents the m over z value between 2000DA and 4000DA. Similarly, B, C, D, E, F, and G represent the region of 4000DA to 6000DA, 6000DA to 8000DA, 8000DA to 10000DA, 10000DA to 12000DA, 12000DA to 14000DA, and 14000DA to 15000DA, whereas the total region means the area between 2000DA and 15000DA.

### Results of comparison between our method and other methods

According to Cruz-Marcelo, Guerra et al. [7], the algorithms PROcess [9] and MassSpecWavelet [12] gave the best performance in terms of peak quantification and peak detection. The authors then suggest a combination of PROcess for peak quantification and MassSpecWavelet for peak detection. In addition, SpecAlign [11] is a well-known software package used to handle mass spectrum data. Therefore, we compared our algorithms, HHTMass1 and HHTMass2, with PROcess, MassSpecWavelet, SpecAlign, and a combination of PROcess and MassSpecWavelet [7]. The combination of PROcess and MassSpecWavelet is abbreviated to PROMSW in this study.

**Figure 7:**
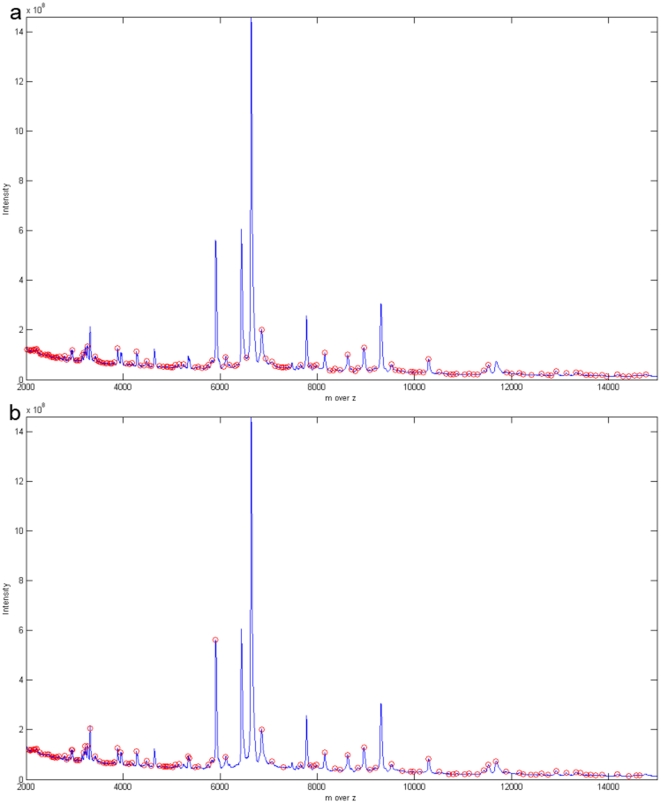
Peaks detected by PRO1 and PRO2. (a) The ovarian cancer dataset preprocessed by PRO1. PRO1 missed the three largest peaks. PRO1 detected 145 peaks in the whole region. (b) The ovarian cancer dataset preprocessed by PRO2. PRO2 misses the two largest peaks. PRO2 detected 114 peaks in the whole region.

**Figure 8:**
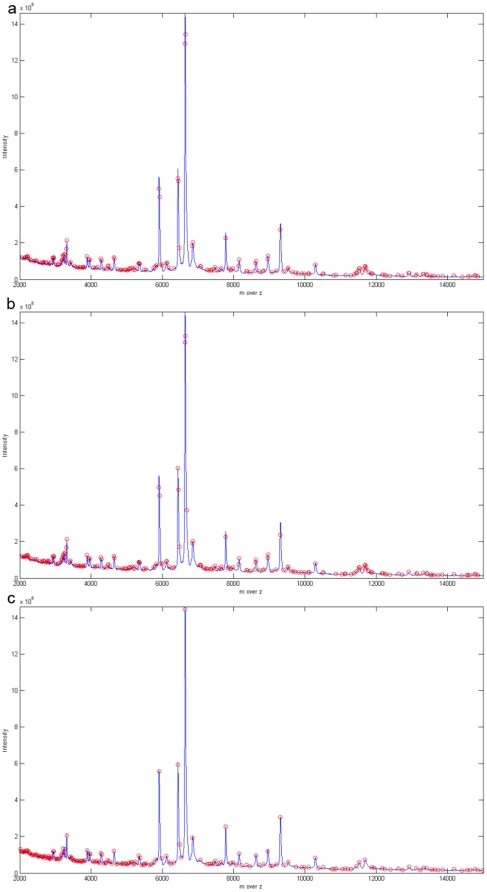
Peaks detected by other methods. (a) The ovarian cancer dataset preprocessed by MassSpecWavelet. MassSpecWavelet produced some redundancies when detecting peaks. For example, the biggest peak is detected twice. MassSpecWavelet detected 188 peaks. (b) The ovarian cancer dataset preprocessed by PROMSW. PROMSW produced some redundancies when detecting the peaks and the result is similar to Figure 8(a). (c) The ovarian cancer dataset preprocessed by SpecAlign. The peaks detected by SpecAlign are concentrated in the region between 2000DA and 6000DA. SpecAlign detected 186 peaks in the whole region.

PROcess used two methods to estimate the baseline. One uses local interpolation and the other uses local regression. Based on Cruz-Marcelo, Guerra et al. [7], the former was designated PRO1 and the latter PRO2. **Table 2** shows that HHTMass1 is able to detect the most peaks and HHTMass2 detects the least peaks. When **Figure 7(a)** and **Figure 7(b)** are examined, PRO1 and PRO2 miss the two most obvious peaks whereas the other algorithms detect these peaks. MSW (**Figure 8(a)**) and PROMSW **Figure 8(b)**) seem to cover most of the obvious peaks on visual assessment. However, as we amplify the spectra, some marked peaks are counted twice with MSW and PROMSW. SpecAlign (**Figure 8(c)**) performs well in terms of visual assessment; however, as can be seen from **Table 2**, SpecAlign detects more peaks than the other approaches in the range between 2000Da and 6000Da. If the associated documents produced by BIO-RAID Laboratories for the Proteinchip matrices (ProteinChip®) are examined, there are three common Proteinchip matrices that are used in SELDI technology, namely:

- Alpha-cyano-4-hydroxycinnamic acid (CHCA), which enables efficient laser desorption and ionization of small proteins (<30 kDa).
- Sinapinic acid (SPA), which enables efficient laser desorption and ionization of larger proteins (10–150 kDa).
- EAM-1, a proprietary formulation, that enables efficient laser desorption and ionization of glycosylated proteins and proteins in the 15–50 kDa range

**Table 2:** The amount of the peaks detected in several popular algorithms.

		Region						
Algorithm	All	A	B	C	D	E	F	G
PRO1	145	46	24	19	18	16	16	6
PRO2	114	40	23	8	12	13	14	4
HHTMass3 (HHT+PRO)	108	21	18	19	13	14	13	10
MSW	188	51	39	25	24	22	20	7
HHTMass1(HHT+MSW)	218	35	32	27	21	44	38	21
PROMSW	198	54	37	33	25	23	18	8
SpecAlign	186	67	43	21	18	16	14	7
HHTMass2(HHT+Specalign)	80	18	17	6	11	8	9	3

PRO1 and PRO2 mean different way to estimate the baseline in PROcess. MSW is the abbreviation of MassSpecWavelet. PROMSW is the combination preprocessing method of PROcess for peak quantification and MassSpecWavelet for peak detection. HHTMass1, HHTMass2, HHTMass3 only replace the preprocess method and use the original peak selection method respectively.

As suggested by the manufacturer's documents, low regions such as 2000Da to 6000Da are usually ignored due to the large amount of noise and the restrictions caused by the Proteinchip matrices and samples. Therefore, when looked at from either the statistical point of view [6], or the biological point of view, the peaks located in the this low region are less significant. As shown in Table 2, SepcAlign has a disadvantage when preprocessing such data and HHTMass is able to conquer this problem. HHTMass1 detects fewer peaks between 2000Da and 6000Da but detects more peaks between 10 kDa and 15kDa. Overall, HHTMass2 is more uniform when examining the low and high regions. This may result to the non-stationary feature if HHT which remove different amount of noises in high and low m/z region.

### Analysis execution time

The major drawback of non-stationary analysis processes as described here is that they takes a longer time for the processing a set of data. For comparison, the experiments using the different methods were carried out on an Intel Pentium 4 personal computer running at 3.0 Gz with 1.5 gigabytes of memory; the computer used Windows XP (c) SP3 and MATLAB(c) R2006a. The dataset used in this test was the MASCAP plasma sample numbers P1∼P5 [22], available at http://www.unich.it/proteomica/bioinf. Each sample contains mass data from about 500DA to 22200DA. Table 3 shows the different times needed for the different approaches, such as wavelet method (MASCAP) and HHT. It is clear that HHT is about 500× slower than wavelet methods such as MASCAP. Using HHT, the user will have to wait more than 1 hour before the analysis is finished but a linear method like MASCAP takes less than 9 seconds. Processing time is an issue that is being addressed at the moment, but it is unlikely that any changes instituted will result in HHT being as fast as the linear methods. Although the longer processing time is a drawback, biologists using mass spectrometry for diagnosis are more interested in accuracy and the processing time at present is not extraordinary (such as days). Methods such as using GPU to accelerated the computation in [23] can easily be reduced (also by improving the computer in terms of CPU, memory and operating system), but a better result is still a better result whether it takes 1 hour or 10 minutes.

**Table 3:** CPU time of different approach (in seconds).

Algorithm Sample No.	HHT	MASCAP (wavelet)
Plasma P_1	4084.7777	8.724561
Plasma P_2	3949.6833	8.996664
Plasma P_3	3908.3151	8.151383
Plasma P_4	4049.2662	7.785374
Plasma P_5	3939.0311	8.117013
Average	3986.2147	8.4094196

### Validation using experimental data

**Figure 9:**
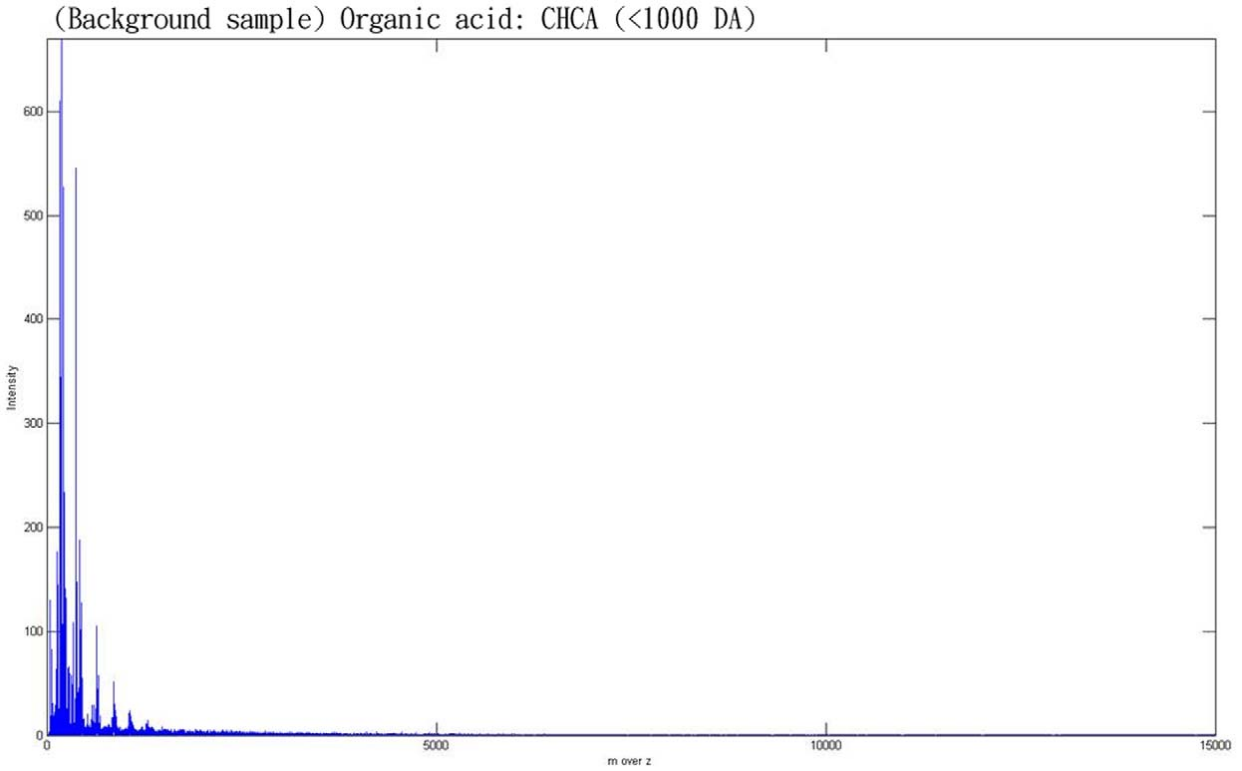
The spectrum of background. The organic acid used in the experiment is CHCA, which has a molecular weight of less than 1000 Da.

**Figure 10:**
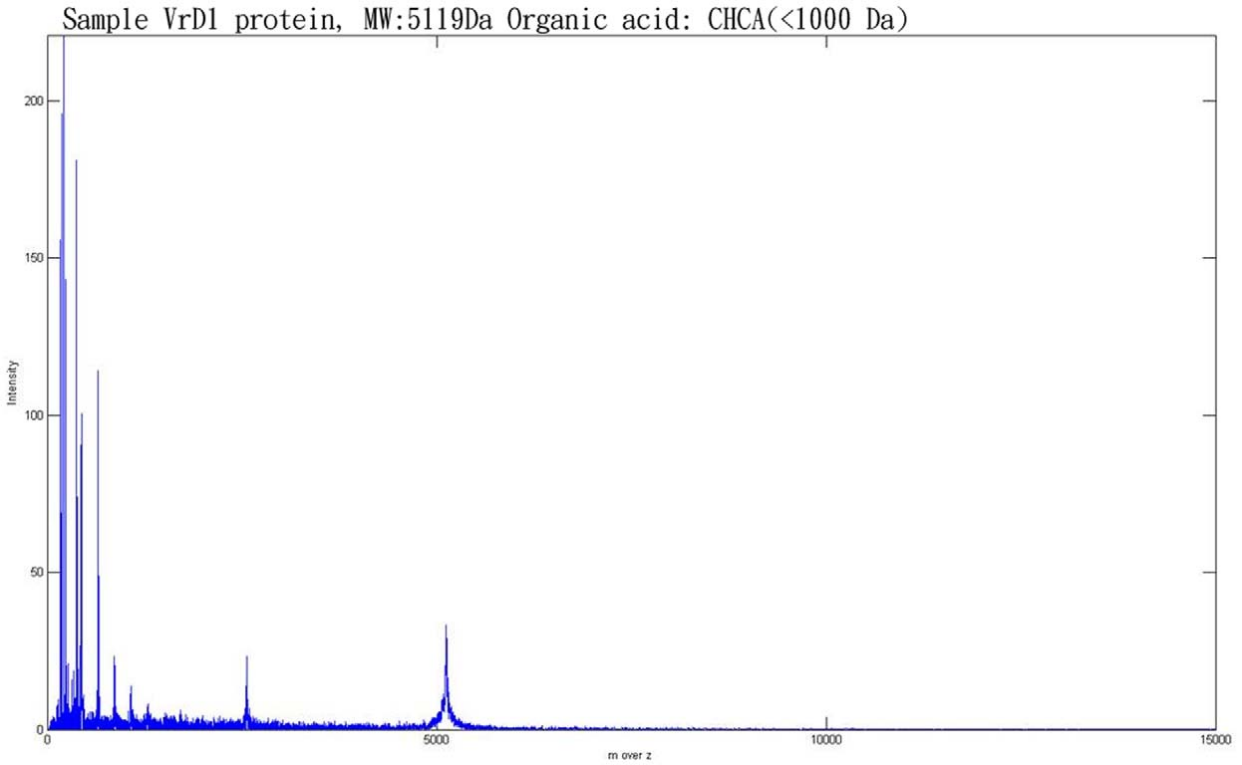
The spectrum of VrD1. VrD1 is a small protein with a molecular weight of about 5119 Da.

**Figure 11:**
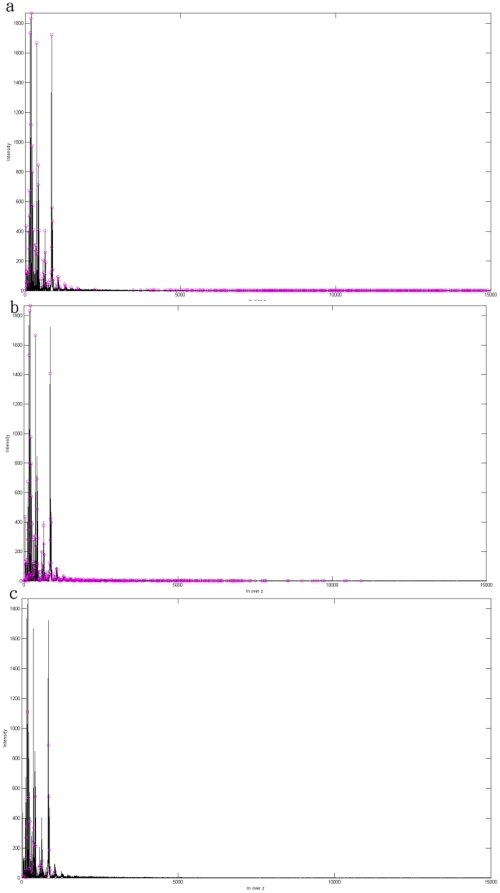
Peak detection on VrD1 data. (a) Peak detection of VrD1 by MassSpecWavelet. The circled purple points are the peaks detected by MassSpecWavelet. (b) Peak detection of VrD1 by SpecAlign. The circled purple points are the peaks detected by SpecAlign. (c) Peak detection of VrD1 by HHTMass2. The circled purple points are the peaks detected by HHTMass2.

**Figure 12:**
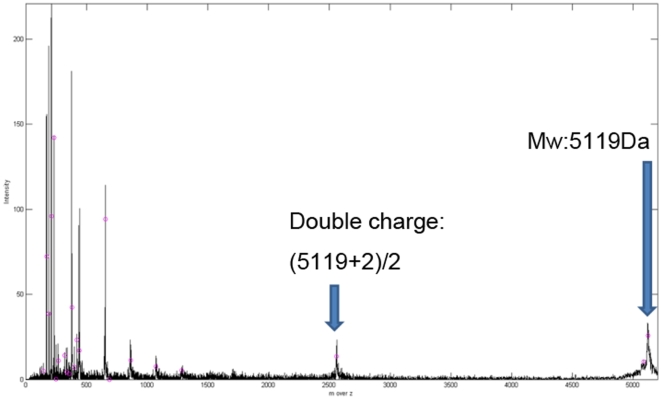
HHTMass on the VrD1 data. HHTMass2 detected the most significant peaks of VrD1, namely the ones at 5119 Da and 2560 Da.

The quality of the peaks ins more important than the number of peaks, but we did not know which peak is correct in the current data. To show that known protein can be detect in our method, we proceed with biology experimental data validation (which did not done in many method discussed in related works). We acquired experimental data from Cathay General Hospital. The dataset consists of two main parts. The first sample contains only water and the molecular weight of water is approximate 18Da, which is closed to zero. The second sample contains the protein, VrD1, which has a molecular weight of about 5119 Da. In both experiments, we use the same organic acid CHCA, which has a molecular weight of less than 1000 Da. **Figure 9** shows the spectrum obtain from the water sample and this is distributed almost completely across a region that is less than 1000 Da. **Figure 10** shows the spectrum of VrD1, which has several peaks that are larger than 1000 Da. However, some noise exists in **Figure 9** because the molecular weight of CHCA is less than 1000 Da. Moreover, if we consider VrD1 with the double charge, then this would create a significant peak located on (5119+1)/2 Da. The major peaks larger than 1000 Da therefore should be at approximate 5119 Da and 2560 Da and the rest of the peaks can be considered to be noise in the VrD1 spectrum. If we use the region of the VrD1 spectrum larger than 1000 Da, MassSpecWavelet detects 340 peaks as shown in **Figure 11(a)** and SpecAlign detects 355 peaks as shown in **Figure 11(b)**. However, HHTMass2 detects only 22 peaks as shown in **Figure 11(c)**. Using this dataset, the most significant peaks are closed to 5119 Da and 2560 Da. HHTMass2 detects the correct significant peaks as shown in **Figure 12**. However, while MassSpecWavelet and SpecAlign are also able to detect the correct significant peaks, these events are masked because these approaches also detect many more of the redundant peaks, which confuse the results.

## Discussion

The mass spectrometry data exist shifting problem which make the m/z scale not stationary. In this study we describe a novel preprocessing method that is better adapted to mass spectrometry data. We use the Hilbert Huang transformation to remove noise from the data by decomposing the spectrum into sixteen components. It should be noted that the total number of components is not always the same. Then by removing the components, we are able to pinpoint the significant information that is the real data. Finally, we estimate the baseline of the spectrum and remove the baseline.

Two peak detection methods, HHTMass1 and HHTMass2, are proposed here. When these are compared, HHTMass1 detects the most of the peaks. Although the number of peaks detected by PROMSW is close to that of HHTMass1, the peaks detected by PROMSW show too much redundancy. HHTMass2 detects the least peaks but these peaks are the most significant peaks. Although the results from SpecAlign are similar to HHTMass2, this program detects too many peaks in the low region of the spectrum, which is generally regarded as a region full of noise. Based on these comparisons, we conclude that using the Hilbert Huang transformation as a preprocessing step and carrying out the relevant modifications to the spectrum is able to improve peak detection performance. The two preprocessing methods, HHTMass1 and HHTMass2, have different advantages and therefore the choice of peak detection method can be split into two distinct policies. Firstly, HHTMass1 is suitable for constructing a model that is able to distinguish a disease group from control group. Secondly, HHTMass2 is suitable for picking out significant biomarkers. In terms of the experimental validation, the use of HHTMass2 decreases redundancy and noise when we used a known protein as a marker, which helped us to separate the important peaks in the spectrum from the noise. In contrast, MassSpecWavelet and SpecAlign tended to detect all peaks present in the sample whether the peaks are significant or not. Our preprocessing approach fits both MALDI and SELDI spectra and using our approach, even if the noise varies between different diseases or between different machines, HHTMass2 is able to ignore these effects.

The preprocessing method used is quite important when analyzing mass spectrometry data because these datasets suffer from unknown amounts of interference from chemical and electrical noise. In addition, in many cases the scale of the spectrum tends to be over large. This increases the difficulty that biologists encounter when analyzing these dataset and constructing a statistical model. The use of a preprocessing method is able to help distinguish the control group from the disease group more easily and increases the accuracy of identifying the correct biomarker.

## References

[1] Cho WC (2007). Proteomics technologies and challenges.. Genomics Proteomics Bioinformatics.

[2] Cho WC, Cheng CH (2007). Oncoproteomics: current trends and future perspectives.. Expert Rev Proteomics.

[3] Salmi J, Nyman TA, Nevalainen OS, Aittokallio T (2009). Filtering strategies for improving protein identification in high-throughput MS/MS studies.. Proteomics.

[4] Shin H, Markey MK (2006). A machine learning perspective on the development of clinical decision support systems utilizing mass spectra of blood samples.. J Biomed Inform.

[5] Hilario M, Kalousis A, Pellegrini C, Muller M (2006). Processing and classification of protein mass spectra.. Mass Spectrom Rev.

[6] Kwon D, Vannucci M, Song JJ, Jeong J, Pfeiffer RM (2008). A novel wavelet-based thresholding method for the pre-processing of mass spectrometry data that accounts for heterogeneous noise.. Proteomics.

[7] Cruz-Marcelo A, Guerra R, Vannucci M, Li Y, Lau CC (2008). Comparison of algorithms for pre-processing of SELDI-TOF mass spectrometry data.. Bioinformatics.

[8] Fung ET, Enderwick C (2002). ProteinChip clinical proteomics: computational challenges and solutions.. Biotechniques.

[9] Li Xea, In Gentleman Rea, editor (2005). Seldi-tof mass spectrometry protein data.. Bioinformatics and Computational Biology Solutions Using R and Bioconductor.

[10] Malyarenko DI, Cooke WE, Adam BL, Malik G, Chen H (2005). Enhancement of sensitivity and resolution of surface-enhanced laser desorption/ionization time-of-flight mass spectrometric records for serum peptides using time-series analysis techniques.. Clin Chem.

[11] Wong JW, Cagney G, Cartwright HM (2005). SpecAlign–processing and alignment of mass spectra datasets.. Bioinformatics.

[12] Du P, Kibbe WA, Lin SM (2006). Improved peak detection in mass spectrum by incorporating continuous wavelet transform-based pattern matching.. Bioinformatics.

[13] Randolph TW, Yasui Y (2006). Multiscale processing of mass spectrometry data.. Biometrics.

[14] Meuleman W, Engwegen JY, Gast MC, Beijnen JH, Reinders MJ (2008). Comparison of normalisation methods for surface-enhanced laser desorption and ionisation (SELDI) time-of-flight (TOF) mass spectrometry data.. BMC Bioinformatics.

[15] Beyer S, Walter Y, Hellmann J, Kramer PJ, Kopp-Schneider A (2006). Comparison of software tools to improve the detection of carcinogen induced changes in the rat liver proteome by analyzing SELDI-TOF-MS spectra.. J Proteome Res.

[16] Wu ZH, Huang NE, Long SR, Peng CK (2007). On the trend, detrending, and variability of nonlinear and nonstationary time series.. Proceedings of the National Academy of Sciences of the United States of America.

[17] DiMagno EP, Corle D, O'Brien JF, Masnyk IJ, Go VL (1989). Effect of long-term freezer storage, thawing, and refreezing on selected constituents of serum.. Mayo Clin Proc.

[18] Qu Y, Adam BL, Thornquist M, Potter JD, Thompson ML (2003). Data reduction using a discrete wavelet transform in discriminant analysis of very high dimensionality data.. Biometrics.

[19] Coombes KR, Morris JS, Hu J, Edmonson SR, Baggerly KA (2005). Serum proteomics profiling–a young technology begins to mature.. Nat Biotechnol.

[20] Morris JS, Coombes KR, Koomen J, Baggerly KA, Kobayashi R (2005). Feature extraction and quantification for mass spectrometry in biomedical applications using the mean spectrum.. Bioinformatics.

[21] Ge G, Wong GW (2008). Classification of premalignant pancreatic cancer mass-spectrometry data using decision tree ensembles.. BMC Bioinformatics.

[22] Mantini D, Petrucci F, Pieragostino D, Del Boccio P, Sacchetta P (2010). A computational platform for MALDI-TOF mass spectrometry data: application to serum and plasma samples.. J Proteomics.

[23] Hussong R, Gregorius B, Tholey A, Hildebrandt A (2009). Highly accelerated feature detection in proteomics data sets using modern graphics processing units.. Bioinformatics.

